# Delayed introduction of the birth dose of Hepatitis B vaccine in EPI programs in East Africa: a missed opportunity for combating vertical transmission of Hepatitis B

**DOI:** 10.11604/pamj.supp.2017.27.3.11544

**Published:** 2017-06-22

**Authors:** Bongomin Bodo, Oliver Ombeva Malande

**Affiliations:** 1 Department of Paediatrics, Faculty of Medicine, Gulu University, Uganda; 2 The East Africa Centre for Vaccines and Immunization (ECAVI), Kampala, Uganda; 3 Department of Pediatrics, Faculty of Health Sciences, Egerton University, Nakuru, Kenya

**Keywords:** Hepatitis B, Expanded Program for Immunization (EPI), East Africa

## Abstract

Vertical Transmission of hepatitis B virus is a major route through which children acquire Hepatitis B infection. Only 10 out of 47 countries in Africa, and none from East Africa; have implemented the WHO recommendation of introducing a birth-dose of hepatitis B vaccine in their EPI program. This article therefore examines the challenges as well as the opportunities that exists for the introduction of a birth-dose of hepatitis vaccine in the National Expanded Program for Immunization (EPI) program by countries in the East African Region. It explores probable health systems factors that have hindered the countries from introducing the birth dose of hepatitis B vaccine and proposes actions that countries can take to introduce the vaccine based on their context by drawing on the experience of some Asian countries.

## Commentary

### The burden (prevalence) of hepatitis B infection in Africa

Hepatitis B virus infection is a viral infection that affects the liver with acute manifestation of symptoms; that in a proportion of people leads to chronic infection; a precursor to the development of serious conditions such as liver cirrhosis and liver cancer (hepatocellular carcinoma). The World Health Organization (WHO) estimates that worldwide, there are currently up to 240 million people living with chronic hepatitis B virus infection, most of whom are in low and middle income countries [1]. The global prevalence of hepatitis B varies widely between countries as well as between population sub-groups. It has been estimated that the prevalence of HBV infection is about 8% in the Western part of Africa, while in the Southern, Central and Eastern parts of Africa slightly lower estimates of 5-7% have been found [2]. It however appears that the prevalence of hepatitis B infections varies according to the sub-group of the population (and the associated risk factors) [3], the HIV prevalence among the individuals studied [4, 5], the age group studied as well as the tests used to assess for the hepatitis B infection [3]. Data from individual East African studies and from Uganda in particular shows even higher prevalence compared to the pooled global estimates stated above [6–8]. A recent population- based study in Gulu district in the northern part of Uganda [7], found a high prevalence of hepatitis B infection at 17.6% among the general population of adults and children. A related hospital- based study among women in antenatal clinics in two big hospitals in the same area of Gulu district [8], also found high prevalence of hepatitis B infection at 11.8%. Important to note in the above two studies in Gulu district in Uganda is the fact that either hepatitis B surface antigen alone or both anti-hepatitis B core antibodies and hepatitis B surface antigen tests were used as markers of hepatitis B infection; and that none of the two studies tested for occult infection using hepatitis B DNA-PCR test. It is therefore possible that the estimates generated by these two studies above may actually be slightly lower than the true estimates if hepatitis DNA-PCR testing were to be included in the tests used, as has been shown in studies elsewhere [9].

### Transmission and control of hepatitis B virus infection

Hepatitis B infection in children may be mainly acquired by two routes: vertical transmission from their mothers and through exposure to other body fluids from close contact with members of the same household, although spread through other means is not unusual. Before the advent of massive early childhood vaccination, clustering of infection and intra-household spread of hepatitis B was not uncommon, and was described in a South African cohort [10]. However after the introduction of large scale vaccination against hepatitis B among children, infection in children through exposures to body fluids of household contacts seems to have reduced in regions of high vaccination coverage and vertical route of transmission from mother to child now appears to be the predominant route [11]. Vertical transmission is more likely where the World health Organization (WHO) recommended dose of hepatitis B vaccine or hepatitis B immunoglobulin is not given at birth to babies born to mother who are hepatitis B surface antigen positive [12]; and more so if they are hepatitis B e-antigen (HBeAg) positive [9, 13, 14]. Following World Health Organization (WHO) recommendation, by 2012 about 181 countries had already introduced HBV vaccine into their National Expanded Program on Immunization (EPI) with reduction in HBV transmission noted in some countries with high burden of HBV infection in Asia. However, with childhood vaccination coverage at about 79%, there is still need to scale it up to over 90% in order to completely stop transmission among children [1]. Among HIV positive pregnant women co-infected with HBV, use of tenofovir and Lamivudine-containing antiretroviral regimens has shown promise as a way of preventing perinatal transmission of hepatitis [15, 16]. However the logistical issues involved as well as ensuring adherence to the medications remain a challenges for resource poor settings such as the countries of East Africa; especially if applied outside the setting of combination antiretroviral treatment for HIV-Hepatitis B co-infection. Access to treatment for chronic hepatitis for the general population (outside the setting of Hepatitis-HIV co-infection) still remains low in developing countries of East Asia and Sub-Saharan Africa that shoulder the global burden of the disease [1]. It is hoped that with the release of WHO guidelines for treatment of chronic hepatitis B infection in early 2015 [12], access to treatment in these high burden countries is likely to improve. Considerable work however still remains as many of the countries have no clear estimates of disease prevalence, have no national policies for hepatitis B control, lack expertise to train health workers on hepatitis B treatment and have weak infrastructure and inadequate human resource to deliver treatment [1]. An even bigger gap in the control of hepatitis B in the East African Region by the member countries appears to lie in the failure to stump out vertical transmission through the introduction of the dose of hepatitis B vaccine given at birth. In a recent review of the status of introduction of the birth dose of hepatitis B in the African region, it was found that only 10 out of the 47 countries had introduced it in their national immunization program; implying that there is ongoing perinatal transmission in most countries, including those in the East African Region [17].

### Challenges and opportunities of introducing the birth dose of hepatitis B in the EPI program in the East African Region

In 2009 the World Health Organization released a position paper giving guidance on the introduction of dose of hepatitis vaccine at birth into the routine EPI program by countries [18]. It was envisaged that by providing guidance to countries, this would translate into introduction of a birth (additional) dose of hepatitis B vaccine to the three doses currently already being given at 6, 10 and 14 weeks of life. However over seven years down the road, of the 5 East African Countries (Uganda, Kenya, Tanzania, Burundi and Rwanda), are all yet to introduce the birth dose of the hepatitis B vaccine into their routine national childhood immunization programs [17]. This is in contrast to intervention for prevention of mother-to-child transmission of HIV (PMTCT) also delivered at birth, which is being fully implemented by all these countries. Recent estimates indicate that a 58% reduction in the perinatal transmission of HIV transmission was achieved between 2001 and 2013 [19]. A big contrast can be drawn between HIV AIDs and hepatitis; two diseases that are big contributors to the Disability Adjusted Life Years (DALYs) lost, though HIV contributing far more bigger DALYs of about 1184 per 100,000 which far outweighs hepatitis B’s contribution which stands at 64 Per 100,000 of DALYs lost according to the global burden of disease study 2010 [20]. From this perspective, it is therefore easy to understand why hepatitis prevention and control has not attracted the kind of attention and consequently funding from major donors as compared to the amount given to HIV/AIDS over the years. However mortality wise, hepatitis is very much a significant contributor to mortality and its contribution to mortality over the years continues to increase as evidenced by comparison of statistics from 1990 and in 2010 [20]. A number of reasons can be advanced for the slow adoption of the WHO recommendations on the introduction of the birth dose of hepatitis into the national EPI programs by member countries. Possible explanation for the above situation could be related to, financing, different delivery platform for the vaccine given at the time of birth in the labour ward and the logistical challenges encountered; including extra cold chain requirements for the hepatitis B vaccine among others [18, 21]. Over the past few years, increasing number of additional vaccines: Human Papilloma Virus (HPV) vaccine, Oral Rotavirus Vaccines (ORV) and the switch to inactivated polio vaccines (IPV) have been recommended by the WHO for introduction into the EPI program by countries [22, 23]. The amount of the funding mechanism which is mainly through the Global Alliance for Vaccine and Immunization (GAVI) has however remained the same; if somewhat even decreased for some countries as they are weaned off GAVI support. Countries are therefore at odds regarding how to prioritize the available funding against other many vaccines that need to be introduced into their EPI programs. While the option of targeting only babies born to Hepatitis B surface Antigen positive mothers for countries could be considered, the added cost of the testing reagents needed and the skilled expertise required for the testing makes this option financially costly as well. Currently the hepatitis B vaccines is given along with other EPI vaccines at 6, 10 and 14 weeks by most countries as is recommended by the World Health Organization [13]. Introduction of an additional dose of hepatitis B vaccine given immediately after birth would require a very close collaboration between the maternity department where the delivery takes place and the staff working in the immunization program; that in most health facility set up, is located in the young child clinic where vaccination usually takes place. In addition since a big proportion of deliveries take place in the community by traditional birth attendants , achieving a high coverage for the birth of hepatitis B vaccines that uses the health facility (labour room) as a delivery platform remains a real challenge in the African Region where health facility delivery remains less than 42% on average [18]. An alternative platform that utilizes community health workers would be needed to bridge this gap if adequate coverage is to be achieved.

Furthermore, considerations for the cold chain in support of introduction of the birth dose of hepatitis B vaccine needs to be made by countries as they consider introducing a birth dose of hepatitis B. The challenge of maintaining a readily available cold chain logistics especially at the primary care level remains a concern in most countries in East Africa [17]. Introduction of the birth dose of hepatitis B would therefore add further strain to the system since a separate system for cold chain for maternity / labour ward for deliveries that occur at hours outside the working days and time for routine immunization would be needed. It would be even more difficult to implement the birth dose of the hepatitis B vaccine in areas where access to immunization is only guaranteed through the outreach model, and first contact with the new born baby occurs usually beyond 72 hours, by which time administration of the hepatitis vaccine B to the newborn would probably be not very effective for prevention of transmission that occurs at or immediately after delivery [17]. A new strategy to address this would need to be devised for maximum impact of the program to be achieved. Despite the challenges mentioned above, success stories from other parts of the world is a clear indication that the birth dose of the hepatitis B vaccine can be successfully introduced in developing countries, provided they maximize on existing resources, use innovative approaches and garner political commitment [21, 24]. Innovative approaches in the administration of hepatitis B vaccines to babies within 24 hours of birth have been reported in China. In one particular study in Hunan province in China, use of out-of cold chain delivery of vaccines to babies born outside the health facilities increased access to hepatitis B vaccine within 24 hours of birth by up to 50%. There was no difference in antibody response to the vaccine even when administered outside the cold chain [24]. This is therefore one strategy that could be considered by countries of the East African Region. Furthermore, to strike the balance between the limiting huge financial investment required to introduce birth dose for every newborn baby in countries with very high fertility rates and the benefit that would accrue from its introduction, a policy of testing all mothers followed by selective administration of the vaccines only to exposed babies could be adopted by East African countries. While this would deviate from current WHO recommendation of universal coverage of the birth dose hepatitis [21, 25], and in addition introduce the costs related to the testing of all mothers, this strategy would help bridge the gap in the interim as countries attempt to mobilize financial resources required to roll out a universal birth dose program for all newborn babies.

In summary, there still remains many barriers to the introduction of the universal dose of hepatitis B vaccine at birth by countries in East Africa. This has hindered the progress towards elimination of vertical transmission of hepatitis B in the East African Region. It is however reassuring to note that experience from China and other Asian countries shows that this goal can be achieved through innovative approaches and use of available research evidence. East African countries can learn from this experience. Indeed the vision and hope of ending vertical transmission of hepatitis B with this current generation; even in resource limited setting such as East Africa is real and with commitment can be realized through innovation and proper prioritization of the available financial resources.

## Competing interests

The authors declare no competing interest.
